# The Prevalence of Juvenile Huntington's Disease: A Review of the Literature and Meta-Analysis

**DOI:** 10.1371/4f8606b742ef3

**Published:** 2012-07-20

**Authors:** Oliver Quarrell, Kirsty L O'Donovan, Oliver Bandmann, Mark Strong

## Abstract

Juvenile Huntington’s disease (JHD) is usually defined as Huntington's disease with an onset ≤ 20 years. The proportion of JHD cases reported in studies of Huntington’s disease (HD) varies. A review of the literature found 62 studies that reported the proportion of JHD cases amongst all HD cases. The proportion of JHD cases in these studies ranged from 1% to 15%, and in a meta-analysis the pooled proportion of JHD cases was 4.92% (95% confidence interval of 4.07% to 5.84%). Limiting the analysis to the 25 studies which used multiple methods of ascertainment resulted in a similar pooled proportion of 5.32%, (95% confidence interval 4.18% to 6.60%). A small difference was observed when the meta-analysis was restricted to studies from countries defined by the World Bank as high income, that used multiple methods of ascertainment, and that were conducted since 1980 (4.81%, 95% confidence interval 3.31% to 6.58%, n=11). This contrasts with the pooled result from three post 1980 studies using multiple methods of ascertainment from South Africa and Venezuela, defined by the World Bank as upper middle income, where the estimated mean proportion was 9.95%, (95% confidence interval 6.37% to 14.22%). These results, which are expected to be more robust than those from a single study alone, may be helpful in estimating the proportion of JHD cases in a given population. Key Words: Juvenile Huntington’s disease, prevalence, epidemiology

## Introduction

Huntington’s disease (HD) is an autosomal dominant, neurodegenerative disorder with onset usually, but not exclusively, between 35 and 50 years of age^1^ . Onset ? the age of 20 is classified as juvenile onset Huntington’s disease (JHD), which can be further divided into childhood (0-10 years) and adolescent (11-20 years) onset. Although there are many similarities with the adult form of the disease, JHD has a clinically distinct presentation; the dominant motor feature being a parkinsonian type syndrome of rigidity, dystonia and bradykinesia, rather than chorea2, 3, 4 . In addition, childhood cases may also present with cerebellar signs, epilepsy, myoclonus and spasticity5, 6 . Behavioural problems and cognitive decline are also common in JHD4, 6 , with additional features of developmental delay and autism in childhood cases^5^ .

The causative mutation in HD has been identified as an expanded CAG repeat sequence in the first exon of the *HTT *gene 7 . A CAG repeat of ? 40 is unequivocally fully penetrant, with CAG repeats of 36-39 showing reduced penetrance^8^ . JHD is usually associated with a CAG repeat of ? 60 repeats in approximately 50% of cases^9^ , with childhood onset often exhibiting a repeat size of ? 805, 10 . However, consistent with findings in adult cases,CAG repeat size does not always correlate with age of onset in JHD^5^ .

The prevalence of HD is considered to be approximately 4-10 cases per 100,000 in Caucasian populations^ 11, 12, 13, 14 ^but this may be higher^15^ . Such variability may be accounted for in part by differences in methods of ascertainment, prevalence method used (period or point prevalence) and diagnostic/age of onset criteria. Low prevalence rates of less than 1 case per 100,000 have been reported in Japanese populations11, 12 . Well developed medical services in Japan mean under-ascertainment is unlikely^12^_.. _More recently, Warby *et al *(2011)^16^ have summarised prevalence data from around the world and discussed the reasons for low prevalence in some countries. Prevalence in African and American black populations is also considered to be lower than in white populations17, 18 . However this may represent under-ascertainment^12^ , with studies employing more extensive methods reporting equivalent rates^19^ . Juvenile onset HD is considered to be extremely rare, with few clinicians ever seeing more than one case. In 1981, Hayden summarised the prevalence of JHD, expressed as a percentage of the total number of HD cases surveyed^11^ . Presented by country, a mean of 5.7% of HD cases were found to be of juvenile onset, with a range of 1-9.6%. Childhood onset was rarer than adolescent onset, with means of 1.3% and 4.4%, respectively. These findings are consistent with the widely accepted belief that JHD is indeed a rare form of the disease.

Although Hayden’s work has been the first and only summary of the epidemiology of JHD known to the authors, there are several limitations in combining and interpreting the data in this way to obtain an estimate of the proportion of JHD cases. The first concerns the pooling of data from different types of studies, such as those reporting period with those reporting point prevalence. Secondly, data are included that are published in more than one paper. Furthermore, the Hayden report includes studies which, due to differences in country and year of study, report prevalence in populations with different age-sex structures. This potentially introduces a bias; lower life expectancies artificially raise the proportion of cases with juvenile onset as patients with adult onset are more likely to die prematurely from unrelated causes. The aim of the current study is therefore to update and expand Hayden’s work, exploring the impact of study level factors on the estimated proportion of HD cases with juvenile onset.

## Methods

We searched the MEDLINE and EMBASE databases for the period 1981-May 2011. The following search terms were used in the title and abstract field:

Huntington’s AND disease AND Prevalence

Huntington’s AND disease AND Population

Huntington’s AND disease AND Epidemiology*

Huntington’s AND disease AND Incidence

Huntington*AND Prevalence

Huntington*AND Population

Huntington*AND Epidemiology*

Huntington*AND Incidence

Juvenile AND Huntington*

(* indicates searches including unlimited truncations of the target word)

Studies that did not include a defined HD population were excluded. Literature reviews were included if they provided information on the total number of HD cases surveyed. Studies were excluded if they did not include information on ages of onset, number of juvenile cases, or patients with onset before age 21. We only used published data once. Articles not written in English were excluded, the one exception being the German language Panse study^20^ as it was included in the original Hayden study. For each study, data relating to the total number of HD cases and total number of juvenile cases were extracted. In addition, the number of cases with childhood (onset 0-10 years) and adolescent (11-20 years) was extracted if available. For each study the proportion of HD cases with juvenile onset was calculated and expressed as a percentage.

**Sub-group analyses.** Studies were subdivided based on methodology, year of publication and country studied. Study methodology was defined as either “multiple methods of ascertainment” or “HD roster/clinic population”. Of these, the most accurate estimates of prevalence were considered to be from studies with multiple methods of ascertainment. Therefore, studies meeting this criterion were used for all further sub-analyses. To account for any biases relating to age-based population structure, studies were further divided by economic status as defined by the World Bank^21^ . To account for any time related changes in population structure and to gain an accurate estimate of the current prevalence of JHD, the final sub-group analysis was conducted using studies published between 1980 and 2011.

**Statistical analysis.** Within each sub-group, study results were pooled in a meta-analysis. Proportions were first transformed using the Freeman-Tukey double arc-sin transformation before being combined. A random effects model was assumed for each meta analysis due to the heterogeneity in the study characteristics. For each meta-analysis the I^2^ value is reported as a measure of statistical heterogeneity. Confidence intervals presented for each individual study were computed using the exact binomial method. All analyses were conducted using the “meta” package in R 2.13.1 22 .

## Results

The search criteria produced a total of 1594 articles. Of these, 48 studies met the inclusion criteria. These studies were combined with those reported by Hayden^11^ , increasing the total number of studies to 59. The study by van Dijk *et al* 2 was excluded as this literature survey was specifically searching for articles with JHD cases and did not define a denominator total HD population. Although the aim of the study was to update Hayden's work we also included the paper by Julia Bell 1948 23 .

The pooled data presented in the Cameron and Venters paper^24^ , which originally included data from their own sample (Scotland) plus that of Bickford and Ellison (Cornwall)^25^and Pleydell (Northamptonshire)26, 27 , has been disaggregated to recreate counts in the three study populations. No JHD cases were reported in the Bickford and Ellison paper^25^ and this was therefore excluded. Therefore, only the data from Pleydell26, 27 and Cameron and Venters^24^ are included in our review. Although the counts of JHD cases in the Cameron and Venters paper are not explicitly reported, these were deduced by calculating the number of cases reported in the two Pleydell26, 27studies.

Cases in the Hayden South African study^28^were categorised by ethnicity into white, mixed and black populations recognising that the white South African population has a mortality distribution similar to that in countries listed as high income, whereas the mixed population has a mortality distribution similar to countries in the upper middle income group (no cases were reported in the black population). To avoid double counting of data in the meta-analysis, the overall total for South Africa was not included. This produced a revised total of 62 studies. A summary of these studies is presented in an appendix.

The results of the meta-analysis are presented as a forest plot in Figure 1. The black population data from Hayden^28^ were excluded for purposes of the statistical analysis since there were no HD cases reported. The overall pooled proportion of JHD cases was 4.92% (95% confidence interval (CI) 4.07% to 5.84%), with a range of 1-15%.

**Forest plot of all studies used in this anlysis:**
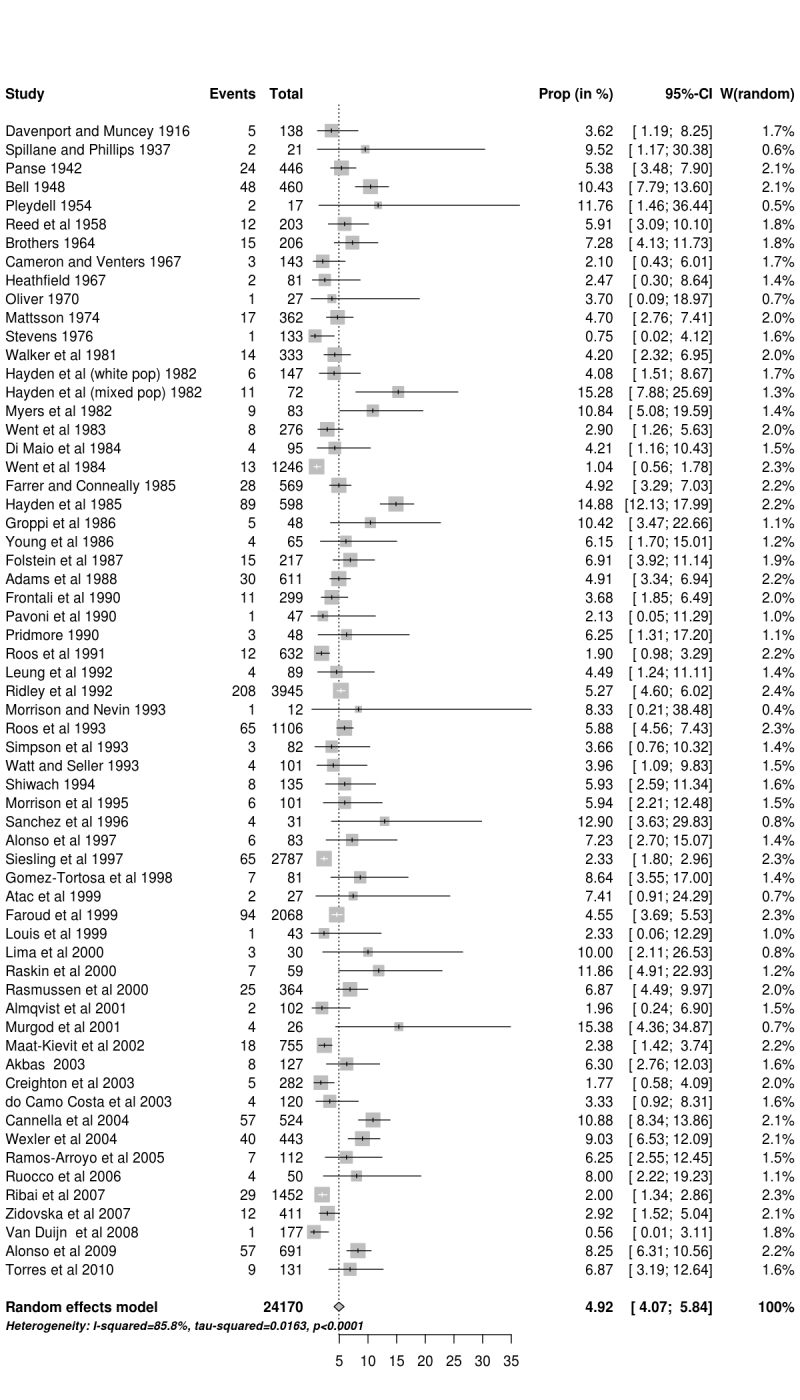


**Sub-analyses. ** Table 1 below summarises the results of a number of sub-group analyses of the data which are described below.

**HD roster or clinic. **Thirty-five studies reported data obtained from either a HD roster or clinic population which are summarised in Fig 2. It is possible that a centre which uses a roster approach may have some patients included in different studies but our final analysis was based on studies which used multiple methods of ascertainment (MMA) so this is is less likely to be a problem.

**Forest plot of studies from a roster or clinic:**
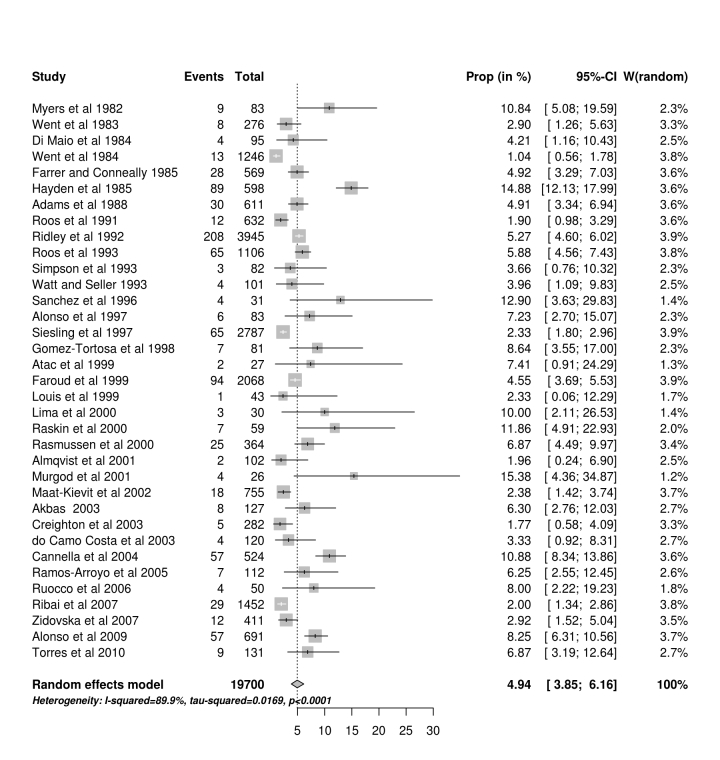

**Multiple methods of ascertainment (MMA).** There were 25 studies applying MMA summarised in Fig 3.

**Forest plot of studies which used multiple methods of ascertainment (MMA):**
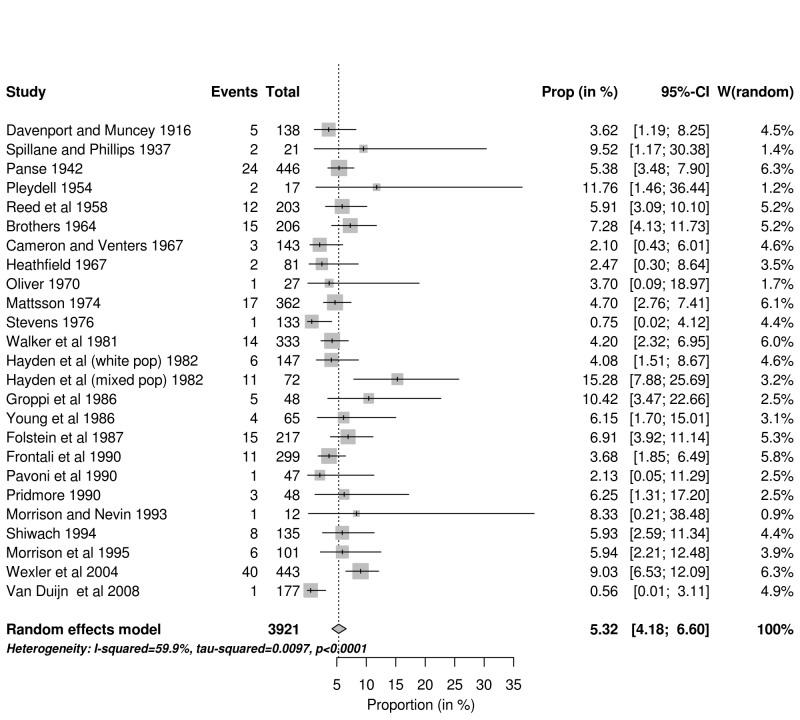


**Income status.** The World Bank^21^ classification of countries was used to subdivide the studies, the majority (22 studies) came from the high income group. By comparison, three studies came from the upper middle income group, two came from Venezuela10, 44 and the third study was that of Hayden^28^, where the data from the White South African population was included in the high income analysis and data from the mixed population was included in the middle income group. Fig 4 summarises these studies from high income countries.

**Forest plot of studies using MMA from high income countries:**
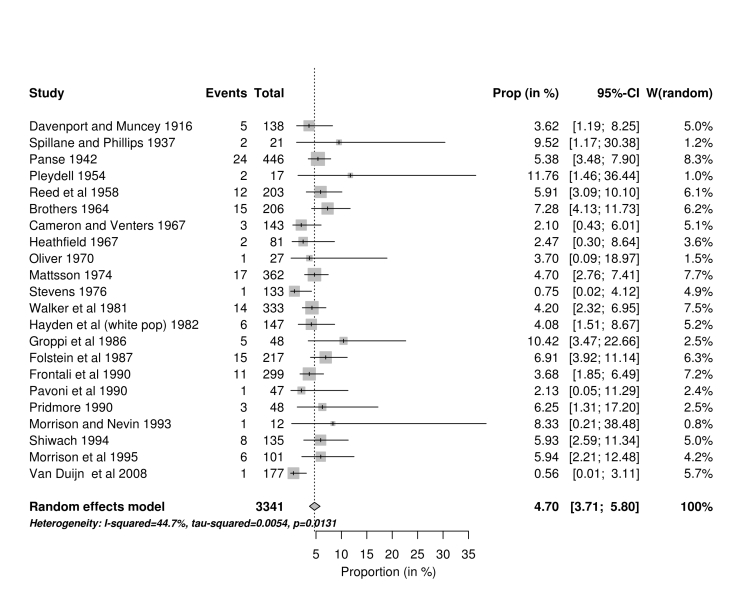

**Post 1980 studies.** Of the MMA studies, 14 were published between 1980 and 2011. Ten were conducted in high income and three in the upper middle income. As above, the final study was that of Hayden^28^, where the data from the white South African population was included in the high income analysis and data from the mixed population was included in the upper middle income group. The forest plot for studies from high income countries is shown in Fig 5 and the forest plot for studies from upper middle income countries is shown as Fig 6.

**Forest plot of post 1980 studies using MMA from high income countries:**
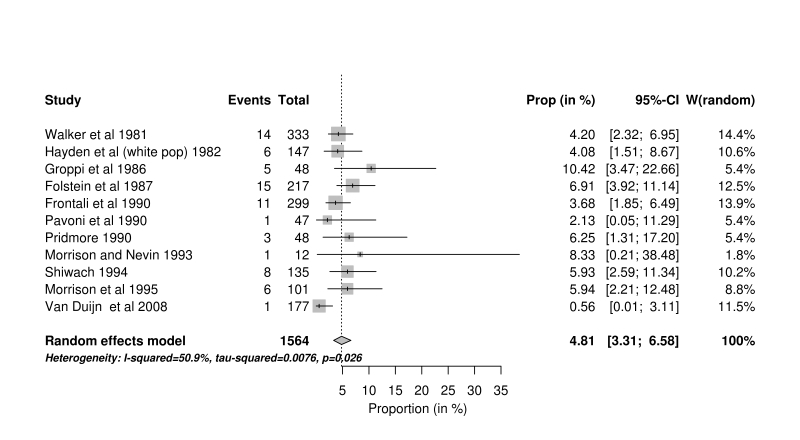

**Forest plot of post 1980 studies using MMA from upper middle income countries:**
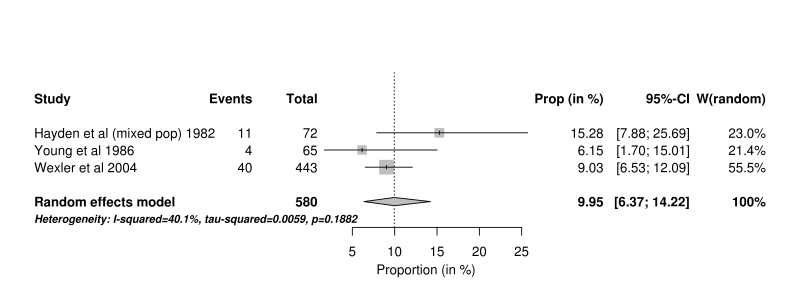

**Table 1:** Summary of Meta-Analyses

Study type	number	Mean %	95% Confidence Interval	Range %
All studies	62	4.92	4.07-5.84	1-15
HD clinic/Roster	35	4.94	3.85-6.16	1-15
Multiple Metods of Ascertainment MMA	25	5.32	4.18-6.60	1-15
MMA + High Income	22	4.70	3.71-5.80	1-12
MMA + Upper Middle Income	3	9.95	6.37-14.22	6-15
MMA + Post 1980 + High Income	11	4.81	3.31-6.58	1-10
MMA + Post 1980 + Upper Middle Income	3	9.95	6.37- 14.22	6-15

Data on childhood and adolescent onset cases was available for 42 studies, representing 475 cases. Of these, 111 (23.4%) and 364 (76.6%) were childhood and adolescent onset, respectively. Only seven studies found a higher proportion of childhood onset cases, with two studies reporting equal numbers. Therefore in 76% of studies, adolescent onset occurred more frequently than childhood onset. When we consider just the most recent studies of the highest quality from the high income group (the MMA-post 1980 studies), the pattern is similar with 40 out of 50 (80%) cases being of adolescent onset.

## Discussion

**Main results.** In the meta-analysis of all the studies identified in this review we estimate the proportion of JHD cases to be 4.92% (95% CI 4.07% - 5.84%). In order to expand on Hayden’s original work^11^ and produce more robust estimates of the proportion of JHD cases, the potential sources of bias associated with the various studies were considered. We identified 35 studies which were based on clinic lists or rosters (Figure 2); and 25 studies which used multiple methods of ascertainment (Figure 3). These gave mean estimates of the proportion of 4.94% and 5.32% respectively. A clinic list or roster approach may downwardly bias the proportion of JHD cases if it is perceived that the clinic serves mainly adult patients. Studies with multiple methods of ascertainment are more likely to give a robust estimate.

Most studies were conducted in Europe and North America so the effect of considering geography was minimal (Figure 4); however, the three studies from economically less developed countries (Figure 5) were effectively from the South African black population and Venezuela. The longitudinal study of families from the area around Lake Maricaibo has been important in that it contributed to the original localisation of the gene to chromosome 4 and for an understanding of the natural history of the condition 10 44 80 The mean proportion of JHD cases from these three studies was 9.95% (95% CI 6.57% - 14.22%). If the age of death was lower in the general population in which these cases live, then those with HD who were destined to develop the condition later in life may not manifest as they die of other causes; consequently the proportion of JHD cases will be higher. In addition, the patients with HD living around Lake Maricaibo are a relatively closed community so it is possible that this also has an effect on the proportion of JHD cases. These three studies were reported after 1980.

Eleven studies were reported after 1980, which used multiple methods of ascertainment, and were from economically more developed countries; these gave a slightly lower proportion of 4.81% (95% CI 3.31% – 6.58%) and may be considered the most robust estimate to use when considering European and North American populations.

**Implications for Research.** At present, there is no treatment to alter the natural history of HD. As soon as treatments become available which do alter the natural history of HD, then it will be important to assess their effects on patients at the more extreme end of the phenotypic spectrum. If a faster rate of disease progression can be demonstrated in this group of patients, then any compound which affects the natural history of HD may show an effect more quickly.

In June 2010, the UK population was estimated to be 62.3 million^79^ . If we assume the prevalence of HD is approximately 4-10 per 100^11^ ^12^ ^13^ ^14^ , in the UK and we also assume some degree of under-ascertainment; therefore, using a figure of 100 HD patients per million would imply that there are around 6,230 patients with HD in the UK, so we should expect to see approximately 300 cases with an onset under the age of 20 years (95% CI 205 – 411). Identifying these patients represents a considerable challenge.

**Conclusion**

We have presented a review of the proportion of cases with JHD from 62 studies. Using data from 25 studies after 1980 which were mainly fromNorthern Europe suggests that the mean proportion of JHD cases is just less than 5%.

## Appendix A

**Summary of all studies used in this analysis:** * refers to studies included in the original Hayden study^11^  The paper from Pleydell 1954^26^ was included with additional data from Pleydell 1955^27^    - means there was no furher specification of age

				0 - 10 years		11-20 years		Total JHD	
Authors	Publication year	Location	Total HD cases	Number	%	Number	%	Number	%
*Davenport & Muncey^29^	1916	USA	138	3	2.2	2	1.4	5	3.6
*Spillane & Phillips^30^	1937	South Wales	21	0	0	2	9.5	2	9.5
*Panse^20^	1942	Germany	446	7	1.6	17	3.8	24	5.4
Pleydell^26^	1954	England	17	0	0	2	11.8	2	11.8
*Reed et al^18^	1958	USA	203	0	0	12	5.9	12	5.9
*Brothers^31^	1964	Australia	206	4	1.9	11	5.3	15	7.3
*Cameron & Venters^24^	1967	Scotland	143	-	-	-	-	3	21
*Heathfield^32^	1967	England	81	0	0	2	2.5	2	2.5
*Oliver^33^	1970	England	115	4	3.5	7	6.1	11	9.6
*Mattsson^34^	1974	Sweden	362	-	-	-	-	17	4.7
*Stevens^35^	1976	England	133	0		1	0.8	1	0.8
Walker et al^36^	1981	South Wales	333	3	0.9	11	3.3	14	4.2
*Hayden et al^28^	1982	S Africa Total	219	6	2.7	11	5	17	7.7
		S Africa Whites	147	3	2.0	3	2.0	6	4.1
		S Africa Mixed	72	3	4.2	8	11.1	11	15.3
		S Africa Blacks	0			0		0	
Myers et al^37^	1982	USA	83	2	2.4	7	8.4	9	10.8
Went et al^38^	1983	Netherlands	276	5	81.	3	1.1	8	2.9
Di Maio et al^39^	1984	Italy	95	-	-	-	-	4	4.2
Went et al^40^	1984	Netherlands	1246	13	1.0	0	0	13	1.0
Farrer & Conneally^41^	1985	USA	569	4	0.7	24	4.2	28	4.9
Hayden et al^42^	1985	USA	598	-	-	-	-	89	14.9
Groppi et al^43^	1986	Italy	48	-	-	-	-	5	10.4
Young et al^44^	1986	Venezueala	65	1	1.5	3	4.6	4	6.2
Folstein et al^19^	1987	USA	217	-	-	-	-	15	6.9
Adams et al^45^	1988	USA	611	5	0.8	25	4.1	30	4.9
Fromtali et al^46^	1990	Italy	299	2	0.7	9	3	11	3.7
Pavomi et al^47^	1990	Italy	47	0	0	1	3.1	1	2.1
Pridmore^48^	1990	Tasmania	48	0	0	3	6.3	3	6.3
Roos et al^49^	1991	Netherlands	632	-	-	-	-	12	1.9
Leung et al^50^	1992	China & Hong Kong	89	4	4.5	0	0	4	4.5
Ridley et al^51^	1992	USA	3945	-	-	-	-	208	5.3
Morrison & Nevin^52^	1993	N Ireland	12	0	0	1	8.3	1	8.3
Roos et al^53^	1993	Netherlands	1106	-	-	-	-	6.5	5.9
Simpson et al^54^	1993	Scotland	82	0	0	3	3.7	3	3.7
Watt & Seller^55^	1993	England	101	-	-	-	-	4	4.0
Shiwach^56^	1994	England	135	0	0	8	5.9	8	5.9
Morrison et al^57^	1995	N Ireland	101	2	2	4	4	6	6
Sanchest et al^59^	1996	Spain	31	0	0	4	12.9	4	12.9
Alonso et al^58^	1997	Mexico	83	-	-	-	-	6	7.2
Siesling et al^4^	1997	Netherlands	2787	-	-	-	-	65	2.3
Gomez-Totosa et al^59^	1998	Spain	81	1	1.2	6	7.4	7	8.6
Atac et al^60^	1999	Turkey	27	0	0	2	7.4	2	7.4
Faroud et al^61^	1999	USA	2068	-	-	-	-	94	4.5
Louis et al^62^	1999	USA	43	0	0	1	2.3	1	2.3
Lima et al^63^	2000	Brazil	30	0	0	3	10	3	10
Raskin et al^64^	2000	Brazil	59	3	5.1	4	6.8	7	11.9
Rasmussen et al^65^	2000	Mexico	364	7	1.9	18	4.9	25	6.9
Almqvist et al^66^	2001	Canada	102	0	0	2	2	2	2.0
Murgod et al^67^	2001	India	26	-	-	-	-	4	15.4
Maat Kievit et al^68^	2002	Netherlands	755	-	-	-	-	18	3.0
Akbas Erginel-Unaltuna^69^	2003	Turkey	127	1	0.8	7	5.5	8	6.3
Creighton et al^70^	2003	Canada	282	-	-	-	-	5	1.8
do Como Costa et al^71^	2003	Portugaul	120	3	2.5	1	0.8	4	3.3
Cannella et al^72^	2004	Italy	524	5	1	52	9.9	57	10.9
Wexler et al^10^	2004	Venezueala	443	-	-	-	-	40	9.0
Ramos-Aroyo et al^73^	2005	Spain	112	-	-	-	-	7	6.3
Ruocco et al^74^	2006	Brazil	50	2	4	2	4	4	8.0
Ribai et al^6^	2007	France	1453	8	0.6	21	1.4	29	2.0
Zidovska et al^75^	2007	Czech Republic	411	-	-	-	-	12	2.9
Van Duijn et al^76^	2008	Netherlands	-	-	-	-	-	1	0.6
Alonso et al^77^	2009	Mexico	691	16	2.3	41	5.9	57	8.2
Torres et al^78^	2010	Peru	131	-	-	-	-	9	6.9
